# Surface Morphology Study of Chemical Vapor Transport of ZnO Crystals

**DOI:** 10.1155/2018/2602596

**Published:** 2018-03-13

**Authors:** Long Fan, Jia Li, Dawei Yan, Liping Peng, Tao Jiang, Xueming Wang, Weidong Wu

**Affiliations:** Research Center of Laser Fusion, China Academy of Engineering Physics, Mianyang 621900, China

## Abstract

A chemical vapor transport (CVT) method was implemented to grow bulk ZnO crystals. X-ray diffraction (XRD), field emission scanning electron microscopy (SEM), and optical microscope (OM) studies were carried out to characterize the surface properties of the grown crystal. The XRD result indicated the exposed solid-vapor interface of the as-grown crystal was composed of (0001) and $\left\{10\overset{-}{1}1\right\}$ faces. Using SEM and OM, we observed small hexagonal pyramids and microstructures formed of crosslines on the as-grown crystal and found hexagonal thermal etching pits on the surfaces of seed crystals. The formation, evolution, and distribution mechanisms of the microstructures were investigated.

## 1. Introduction

Wurtzite ZnO is a direct wide bandgap semiconductor with a bandgap energy of about 3.37 eV at room temperature and has a large free exciton binding energy of about 60 meV [1–3]. It has attracted considerable attention due to its promising applications in laser diodes for blue and UV spectral regions, transparent field effect transistors, and RT polariton lasers. Besides, ZnO single crystals can be used as substrates for the heteroepitaxial growth of GaN, which presents a lattice mismatch less than 1.8%.

To date, bulk ZnO single crystals have been grown via a hydrothermal (HT) method [4–6], pressurized melt method [7, 8], flux method [9–11], and chemical vapor transport (CVT) method [9, 10, 12, 13]. Among them, CVT method is suitable for high-purity ZnO crystals grown at the relatively low temperature. The advantageous feature of this method is the purification effect during vapor transport. Using carbon as the transport agent, several research groups have grown high-quality ZnO crystals successfully. A lot of research has been carried out on the structural, optical, and electrical properties of CVT ZnO crystals [13–17]. However, there is scarce literature dealing with the surface properties and morphology of ZnO crystals grown from the vapor phase. To our knowledge, there is no report of hexagonal pyramids and regular microstructures formed of crosslines on the crystallization front of the CVT ZnO bulk crystals. The shape of crystallization front is known to determine the growth stability of the crystal. Thus, it is important to investigate the morphology and the growth mechanism of growth interface.

In this work, we focus on the microstructures appearing on the surface of a CVT ZnO crystal. X-ray diffraction (XRD), field emission scanning electron microscopy (SEM), and optical microscope (OM) studies were carried out to characterize the surface properties of the obtained crystal. The formation, evolution, and distribution mechanisms of the microstructures were investigated. The conclusions may be helpful for improvement of the growth techniques of CVT ZnO crystals.

## 2. Experiment

**Figure 1:**
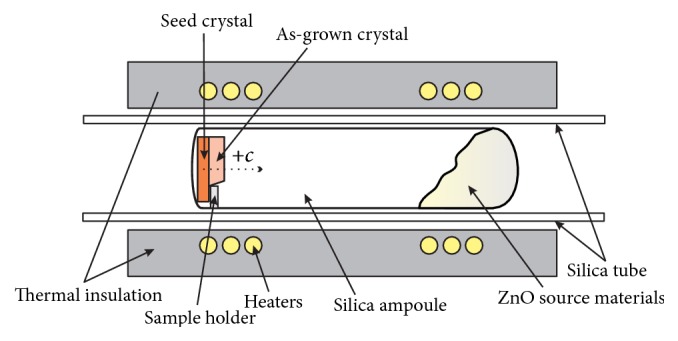
Scheme of the horizontal CVT growth system.

A horizontal CVT system was used for the growth of ZnO single crystals, as shown in Figure 1. Approximately 10 g of ZnO polycrystalline materials with about 0.12 g graphite powder were placed in a fused-silica ampoule and then evaporated to 10^−4^ Pa. After sealing, the ampoule was placed in a horizontal tube furnace. The seed crystal was located at one end of the ampoule, while the source materials were placed at the other end of the ampoule. In our CVT growth system, the exposed seed surface was (0001) face, and a silica sample holder was placed next to the seed crystal to fix the seed. The growth experiments were carried out under the source temperature* Ts* = 1010°C and the growth temperature* Tg* = 980°C. Growth period was 2 weeks.

The crystalline property of the obtained crystal was examined using X-ray diffraction (Philips X'Pert PRO, Cu* Kα* radiation,* λ *= 0.154 nm). The surface morphology of the crystal was characterized using FE-SEM (Quanta 250, FEI) and OM (BX61-32FDIC-S08, OLYMPUS).

## 3. Results and Discussion

**Figure 2:**
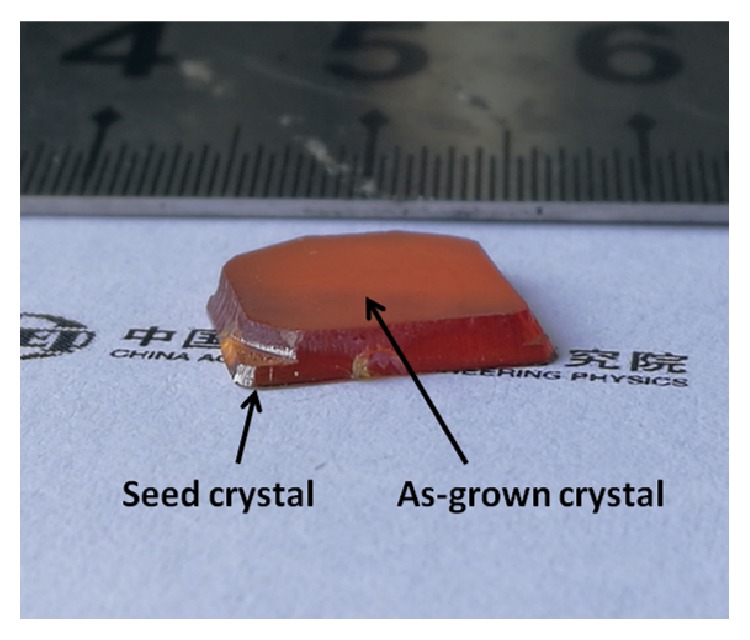
Photograph of obtained crystal ingot consisting of the as-grown crystal and the seed crystal.

**Figure 3:**
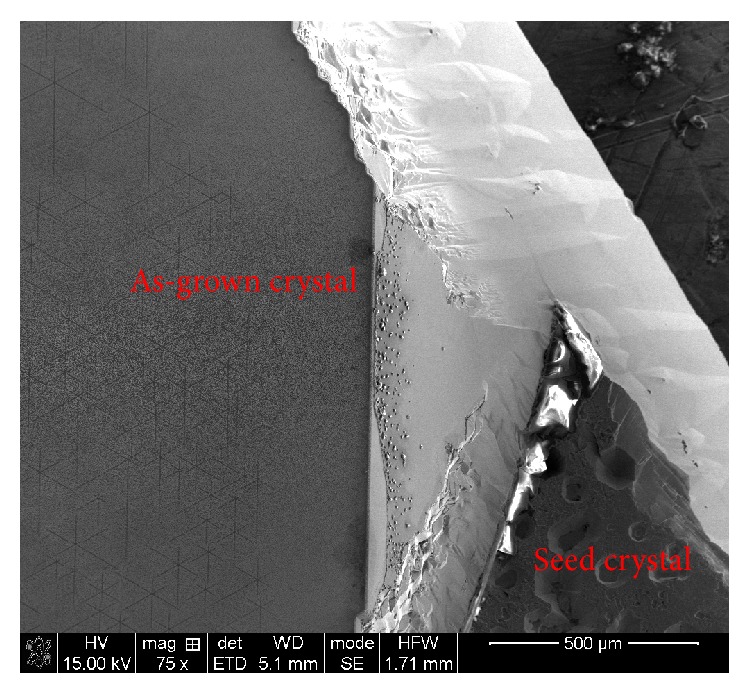
SEM photograph of the area containing both the as-grown crystal and the seed crystal with enlargement of 75 times.

Figure 2 shows the photograph of the obtained crystal ingot, which consists of two parts: the as-grown crystal and the seed crystal.  Figure 3 shows the SEM photograph of the area containing both the as-grown crystal and the seed crystal.

**Figure 4:**
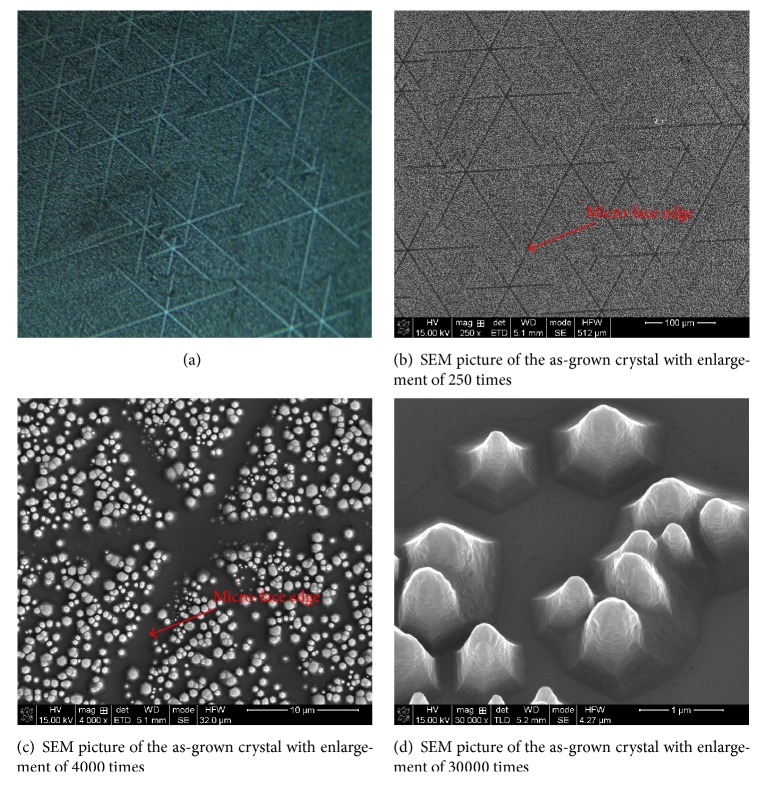


Figures 4(a)–4(d) show OM and SEM photographs of the as-grown crystal with different resolution. The microstructures formed of many crosslines are observed with low resolution. Small hexagonal pyramids are found with high resolution.

**Figure 5:**
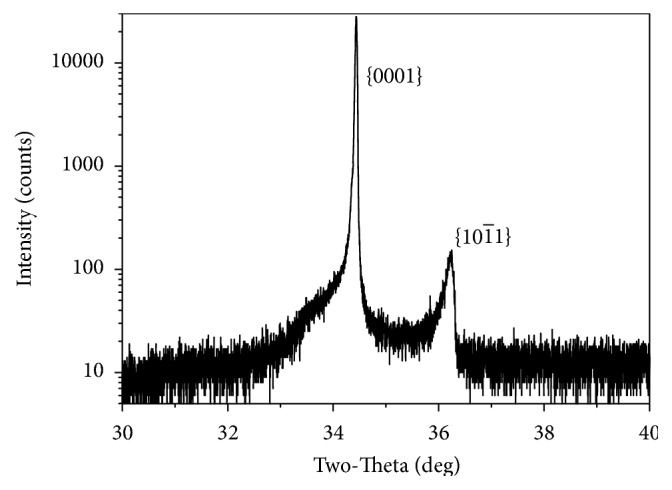
XRD pattern of the as-growth crystal.

The surface of the as-grown crystal was subjected to XRD analysis. The XRD pattern is shown as a logarithmic scale in Figure 5, from which two diffraction peaks are identified to be the crystal face sets {0001} and $\left\{10\bar{1}1\right\}$. The peak observed at about 2*θ* = 34.4° corresponds to ZnO (0001) face, revealing the preferentially oriented growth direction along the *c*-axis. The peak at about 2*θ* = 36.2° indicates some $\left\{10\bar{1}1\right\}$ faces existed on the solid-vapor interface.

**Figure 6:**
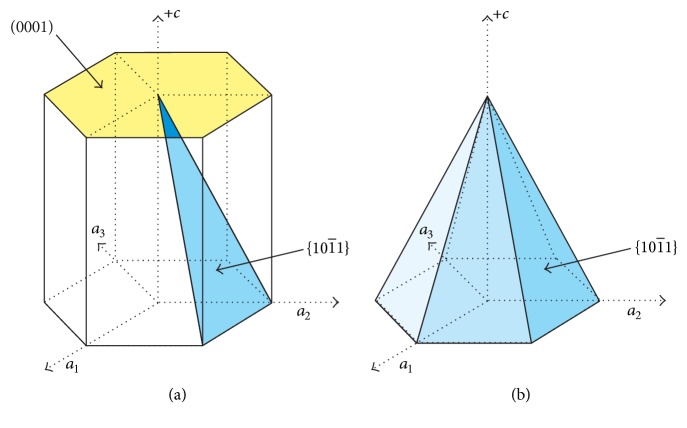
(a) Sketch of ZnO structure highlighting (0001) and $\left\{10\bar{1}1\right\}$ faces. (b) Hexagonal pyramid consisting of $\left\{10\bar{1}1\right\}$ faces and $\left(000\bar{1}\right)$ face.

Figure 6(a) shows a sketch of wurtzite ZnO structure, in which the faces of (0001) and {10-11} are highlighted. A schematic hexagonal pyramid consisting of a $\left(000\bar{1}\right)$ face for the base and $\left\{10\bar{1}1\right\}$ faces for the side surfaces is shown in Figure 6(b). ZnO is structurally described as a series of planes composed of fourfold tetrahedrally coordinated O^2-^ and Zn^2+^ ions stacked alternatively along the *c*-axis [18]. These ions produce the positively charged Zn (0001) plane and the negatively charged O $\left(000\bar{1}\right)$ plane, resulting in the spontaneous polarization along the *c*-axis and the consequential different growth rates of polar surfaces.

For ZnO crystal, it has been observed that the maximal crystal growth velocity is fixed in the 〈0001〉 direction. The relationship between the growth rates towards different directions is found as follows: $V_{\langle 0001\rangle }>V_{\langle \begin{array}{cc} 10 & \overset{-}{1}1 \end{array}\rangle }>V_{\langle \begin{array}{cc} 01 & \bar{1}0 \end{array}\rangle }>V_{\langle \begin{array}{cc} 000 & \bar{1} \end{array}\rangle }$ [19]. Generally, the faces perpendicular to the fast growth direction have small surface areas, while the faces perpendicular to the slow growth direction dominate the final morphology. Because of the differences of growth rates of polar faces, hexagonal pyramid-like ZnO microcrystals are formed after nucleation, which are observed with high resolution in Figure 4(d).

**Figure 7:**
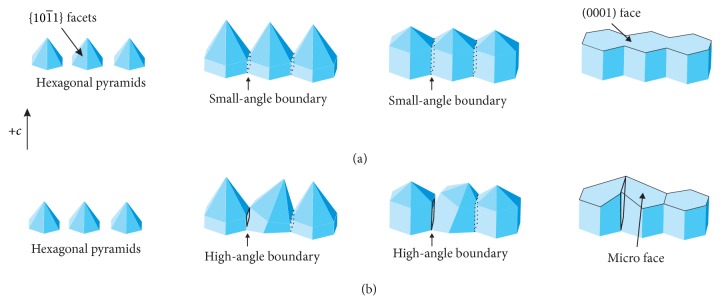
Formation of (a) flat face; (b) micro face on the as-grown crystal.

Based on the observed surface morphology of the as-grown crystal, we proposed possible formation mechanisms of microstructures formed of crosslines observed with low resolution. During the growth process, ZnO nuclei first form on the solid-vapor interface, followed by formations of hexagonal pyramid-like microcrystals. As the pyramids grow and pack closely together, V-shaped interfaces and small-angle boundaries form between them. With increasing growth time, the V-shaped regions are filled up and the small-angle boundaries gradually disappear. The flat (0001) face then appears on the vapor-solid interface, as shown in Figure 7(a). In some cases, the crystal is affected by disadvantageous growth conditions, which may lead to subgrain tilting and boundary elimination and yield high-angle boundaries between subgrains. Micro faces having an angle with respect to the flat plane appear on the surface of solid-vapor interface, as shown in Figure 7(b).

**Figure 8:**
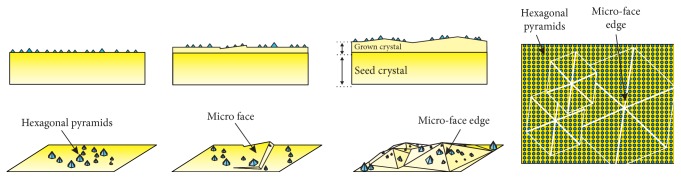
Formation of the crosslines on the as-grown crystal.

With the increase of time, micro faces gather together and gradually form hexagonal growth steps, which contain regular micro-face edges. Then new nuclei and small hexagonal pyramids form continually on the hexagonal growth steps. For hexagonal pyramids are not able to grow on the edges of micro faces, crosslines of vacant space without hexagonal pyramids are formed. The formation of the crosslines is illustrated schematically in Figure 8.

**Figure 9:**
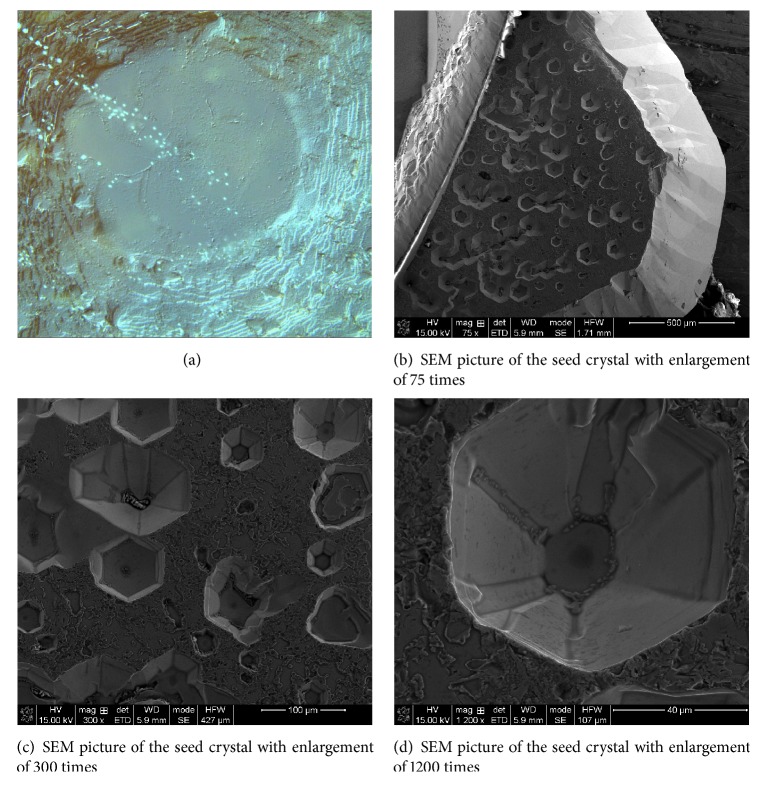


**Figure 10:**
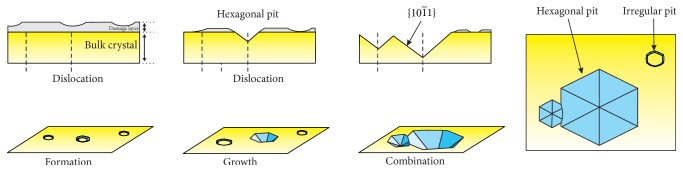
Development of the thermal etching pits on the seed crystal.

Figures 9(a)–6(d) show OM and SEM photographs of the seed crystal. The appearance of thermal etching pits was observed on the seed crystal. Because a part of the seed was shaded from the flow of supersaturated vapor by the sample holder in our CVT system, thermal etching effect occurred at the masked area of the seed crystal. Etching process first occurred at the damage layer above the bulk crystal and yielded irregular etching pits. Once the damage layer is depleted, thermal etching pits were produced at the points of emergence of dislocation lines on the surface of the bulk crystal. Due to the differences in etching rates between the polar faces, the {10-11} planes will appear in the later stage [20]. As a result, hexagonal thermal etching pits composed of six {10-11} planes and the +*C* plane are formed. As the growth time is increased, the size of the etching pits increases, and some pits dissociated and enlarged. The thermal etching mechanism of the seed crystal is illustrated schematically in Figure 10.

## 4. Conclusion

A CVT method was adopted to growth bulk ZnO crystals. Surface properties and microstructures appearing on the surface of the crystal were studied by XRD, OM, and SEM. Hexagonal pyramids and hexagonal thermal etching pits were observed on the as-grown crystal and the seed crystal, respectively. The formation, evolution, and distribution mechanisms of the observed microstructures were investigated.

## References

[1] Huang F., Lin Z., Lin W. (2014). Research progress in ZnO single-crystal: Growth, scientific understanding, and device applications. *Chinese Science Bulletin*.

[2] Ozgur U., Alivov Y. I., Liu C. (2005). A comprehensive review of ZnO materials and devices. *Journal of Applied Physics*.

[3] Triboulet R. (2014). Growth of ZnO bulk crystals: A review. *Progress in Crystal Growth and Characterization of Materials*.

[4] Ohshima E., Ogino H., Niikura I. (2004). Growth of the 2-in-size bulk ZnO single crystals by the hydrothermal method. *Journal of Crystal Growth*.

[5] Wang B., Mann M., Claflin B. (2013). Hydrothermal growth and characterization of aluminum-doped ZnO bulk crystals. *Oxide-Based Materials and Devices IV*.

[6] Zhang C.-L., Zhou W.-N., Hang Y. (2008). Hydrothermal growth and characterization of ZnO crystals. *Journal of Crystal Growth*.

[7] Anwand W., Brauer G., Grynszpan R. I. (2011). Characterization of microstructural defects in melt grown ZnO single crystals. *Journal of Applied Physics*.

[8] Mukhanov V. A., Sokolov P. S., Baranov A. N., Timoshenko V. Y., Zhigunov D. M., Solozhenko V. L. (2013). Congruent melting and rapid single-crystal growth of ZnO at 4 GPa. *CrystEngComm*.

[9] Hong S.-H., Mikami M., Mimura K. (2009). Growth of high-quality ZnO single crystals by seeded CVT using the newly designed ampoule. *Journal of Crystal Growth*.

[10] Hong S.-H., Sato T., Mikami M. (2009). Growth of ZnO crystal by self-flux method using Zn solvent. *Journal of Crystal Growth*.

[11] Xu J., He Q., Shen H., Jin M., Zhang Y., Li X. (2011). Flux Bridgman growth and the influence of annealing temperatures on PL properties of ZnO single crystals. *Crystal Research and Technology*.

[12] Udono H., Sumi Y., Yamada S., Kikuma I. (2008). Crystal growth of ZnO bulk by CVT method using PVA. *Journal of Crystal Growth*.

[13] Wei X., Zhao Y., Dong Z., Li J. Characterization of bulk ZnO single crystal grown by a CVT method.

[14] Auret F. D., Goodman S. A., Legodi M. J., Meyer W. E., Look D. C. (2002). Electrical characterization of vapor-phase-grown single-crystal ZnO. *Applied Physics Letters*.

[15] Hong S., Mun J., Mimura K. (2012). Effect of annealing in an O-2 atmosphere on the electrical properties of high-quality ZnO single crystals grown by seeded chemical vapor transport. *Journal of Cermaic Processing Research*.

[16] Wei X., Zhao Y., Dong Z., Li J. (2008). Investigation of native defects and property of bulk ZnO single crystal grown by a closed chemical vapor transport method. *Journal of Crystal Growth*.

[17] Zhao Y., Zhang F., Zhang R. Native deep level defects in ZnO single crystal grown by CVT method.

[18] Zhou X., Xie Z.-X., Jiang Z.-Y. (2005). Formation of ZnO hexagonal micro-pyramids: A successful control of the exposed polar surfaces with the assistance of an ionic liquid. *Chemical Communications*.

[19] Lu F., Cai W. P., Zhang Y. (2008). ZnO hierarchical micro/nanoarchitectures: solvothermal synthesis and structurally enhanced photocatalytic performance. *Advanced Functional Materials*.

[20] Jo W., Kim S.-J., Kim D.-Y. (2005). Analysis of the etching behavior of ZnO ceramics. *Acta Materialia*.

